# Dexamethasone as Adjuvant Therapy for Bacterial Meningitis in Children: What About *Streptococcus pneumoniae*?

**DOI:** 10.1093/ofid/ofaf456

**Published:** 2025-08-01

**Authors:** Ellen R Wald

**Affiliations:** Department of Pediatrics, University of Wisconsin School of Medicine and Public Health, Madison, Wisconsin, USA

**Keywords:** Bacterial meningitis, children, dexamethasone, steroids, *Streptococcus pneumoniae*

## Abstract

Despite advances in antimicrobial treatment of bacterial meningitis in children, morbidity and mortality are substantial. Adjuvant dexamethasone is currently recommended for children with meningitis due to *Haemophilus influenzae* type b to improve outcome; recommendations for use of dexamethasone in children with meningitis due to *Streptococcus pneumoniae* are controversial. Conclusions regarding dexamethasone use for children with pneumococcal meningitis are derived from existing meta-analyses, which are confounded by the inclusion of studies with small numbers, substantial economic variability, and poor design. In contrast, circumstantial data derived from adult studies of pneumococcal meningitis are compelling in demonstrating effectiveness of dexamethasone in improving mortality and neurologic outcomes. Barring known differences in the inflammatory response stimulated by *S pneumoniae* according to age or the impact of steroids on inflammation according to age, this review endorses dexamethasone for children with pneumococcal meningitis.

Bacterial meningitis remains a serious infection for both children and adults causing substantial morbidity and mortality [1]. Historically, the 3 pathogens causing bacterial meningitis in childhood in the United States (US) in the era preceding the development of conjugate vaccines were *Haemophilus influenzae* type b (Hib), *Streptococcus pneumoniae*, and *Neisseria meningitidis*, in that order. Before the development of the first conjugate vaccine for Hib, this bacterial species was responsible for 20 000 cases of invasive disease each year, including at least 10 000 cases of meningitis, and was the leading cause of acquired cognitive impairment in children in the US. *Streptococcus pneumoniae* and *N meningitidis* together were still less than the number of cases of *H influenzae*—accounting for about 30% of cases or less, depending on the series. Survival in the preantibiotic era was extremely low (≤10%) and always with severe sequelae. Currently, the mortality rate in the US, depending on the specific bacterial species causing infection, is in the range of 7%–10% [2] in children and 15%–37% for adults [3].

## PATHOPHYSIOLOGY OF BACTERIAL MENINGITIS

The bacteria causing childhood meningitis (*S pneumoniae*, *N meningitidis*, and Hib) reside, under usual circumstances, in the nasopharyngeal mucosa. Through events which have not yet been completely delineated, but which are nearly always preceded by a viral upper respiratory infection, a bacteremia occurs, which successfully seeds the meninges. Each of these bacteria has a polysaccharide capsule as its outermost surface, which is important in impeding phagocytosis, thereby eluding an important innate defense mechanism. However, the cell walls of these organisms are distinctly different. Evidence shows that the components of these cell walls are critical in the initiation of inflammation in the cerebrospinal fluid (CSF) but that the inflammatory response is different for gram-positive (*S pneumoniae*) and gram-negative (*H influenzae* and *N meningitidis*) organisms [4, 5]. The major constituents of gram-positive cell walls are teichoic acid and peptidoglycan. In contrast, lipopolysaccharide is a major constituent of the cell wall of *H influenzae.* Different patterns of cytokine induction and tumor necrosis factor release result when these cell walls bind to various cell-surface receptors. Not only do intact cell walls initiate an intense inflammatory response, but the products of bacteriolysis, initiated by the commonly used antibiotic treatment of bacterial meningitis (including ceftriaxone, ampicillin, and vancomycin), are also potent stimulators of the inflammatory response [6].

The sequence of events occurring within the CSF begins with recognition of these cell walls by pattern recognition receptors in the form of Toll-like receptors found on many different cell types including endothelial and glial cells. This is followed by the production of tumor necrosis factor alpha (TNF-α) and interleukin 1-beta (IL-1β), as well as other inflammatory mediators in addition to complement activation, which together initiate a complex series of events including an increase in vascular permeability, ingress of polymorphonuclear leukocytes, and promotion of the coagulation cascade [5 ]. The combination of edema (from vasodilatation and increased permeability) and cellular inflammation combine to increase intracranial pressure and decrease cerebral perfusion [5]. The net impact of this may be a combination of neuronal cell damage and cell death. In some cases, the increase in intracranial pressure can lead to herniation of the brain. At the same time, the orchestrated inflammatory response results in bacterial killing, removal of debris, and hoped-for restoration of tissue integrity. The balance of these activities determines outcome.

One of the mechanisms by which steroids are beneficial as adjuvant therapy is by blunting the inflammatory response initiated by the bacterial cell wall without impairing the immune response necessary for bacterial killing. Studies in the rabbit model of meningitis due to Hib have demonstrated that dexamethasone administration reduces brain edema and intracranial pressure as well as the production of lactate [7]; others have shown a decrease in activity of TNF-α and IL-1β [8]. Studies in children confirm that those treated with dexamethasone had significantly lower concentrations of IL-1β and lactate and higher concentrations of glucose in their CSF than those receiving placebo [9]. Furthermore, steroids are known to stabilize vascular endothelial cell membranes resulting in a reduction of vasodilatation, vascular permeability, and therefore cerebral edema; they also redirect leukocyte migration away from sites of inflammation [10].

## ADJUVANT STEROIDS IN CHILDREN WITH MENINGITIS

The advent of antimicrobials—sulfonamides in the mid-1930s and penicillin in the mid-1940s—led to tremendous progress for patients with meningitis. However, although antibiotic therapy substantially improved outcome, many children and adults bore permanent evidence of their infection. For children, the major neurologic complications include hydrocephalus, epilepsy, hearing loss, vision loss, a variety of motor issues including paresis and ataxia, and significant intellectual deficits. Together these issues affect anywhere from 15% to 40% of those who recover. A more modest intellectual impairment and learning problems affect an additional 10%–20% of children.

### Early Results

Early in the antibiotic era, practitioners began to pose the question as to whether steroids, which became available for clinical use in 1949, might improve the outcome of bacterial meningitis beyond that which was achieved with antibiotics. One study reported in 1957 [11] and 2 others reported in 1969 [12, 13] concluded that steroids did not offer a significant benefit. In these studies, there was no attention given to the timing of administration of the steroid and doses varied. Antibiotic regimens also varied but included penicillin, sulfonamides, and chloramphenicol until ampicillin became available in the 1960s. After the emergence of β-lactamase–producing Hib, chloramphenicol was reintroduced into the therapeutic regimen for meningitis. A very cogent issue identified by these early studies was the critical importance of assessing degree of impairment of children at the outset of treatment to assure comparability of groups when evaluating the outcome of any intervention.

### Later Results

Second- and third-generation cephalosporins became available for clinical use in patients with meningitis in the 1980s. Lebel et al [14] reported a pivotal study in 1988 involving 2 cohorts of children with meningitis. In group 1, the antibiotic treatment was cefuroxime and in group 2, the treatment was ceftriaxone. Children were randomized to receive dexamethasone versus placebo. Only when the results of hearing in the 2 groups were combined were children receiving dexamethasone shown to experience significantly less hearing loss than those receiving placebo. Although, the overall outcomes favored the dexamethasone group, the results did not reach significance in most cases. In particular, the effect was most remarkable for those children with meningitis due to *H influenzae*. However, concerns were raised regarding whether cefuroxime was as effective an antibiotic as ceftriaxone for the treatment of *H influenzae* meningitis. The speculation was that perhaps dexamethasone might appear more effective when used with an antibiotic that was inferior. This concern was substantiated by a report from Schaad et al [15], comparing ceftriaxone and cefuroxime for bacterial meningitis, which did demonstrate the superiority of ceftriaxone regarding more rapid sterilization of the CSF as well as the benefits of less hearing loss.

In 1995, Wald et al [16] reported another prospective, placebo-controlled trial of 173 children with meningitis treated with ceftriaxone from 6 children's hospitals and randomized to receive dexamethasone or placebo. The unique feature of this study was the performance of an initial audio-evoked brain response within the first 24 hours of admission to determine the timing of onset of deafness. While bilateral deafness was more common in placebo compared to dexamethasone recipients, more placebo recipients were deaf at the time of entry to the study. Outcome was similar for placebo and dexamethasone recipients.

A meta-analysis of studies evaluating adjuvant dexamethasone in childhood meningitis published in 1997 reviewed only studies done after 1988 in the hopes of clarifying the impact of dexamethasone on meningitis due to *S pneumoniae* and its effect on neurologic issues other than hearing loss [17]. However, the inclusion of studies in which only children older than 2 or 4 years were either enrolled or had hearing evaluations challenges the likely generalizability of results, as meningitis peaks in children <2 years of age in the US. Ultimately, the authors concluded that (1) dexamethasone had the most remarkable impact on children with Hib regarding hearing loss, (2) the benefit of dexamethasone for meningitis due to *S pneumoniae* was still unknown, and (3) there was a legitimate question of whether limiting dexamethasone to 2 days rather than 4 days might maximize benefit and minimize adverse reactions.

The net outcome of these studies is the statement from the Committee on Infectious Diseases of the American Academy of Pediatrics endorsing the use of dexamethasone for meningitis caused by *H influenzae* but being noncommittal regarding their use for *S pneumoniae* [18]. As *H influenzae* has all but disappeared in the US, this leaves a gap for the recommendation regarding other etiologies of meningitis, in particular, *S pneumoniae*. Furthermore, there is no likelihood that this question can be answered by a prospective trial in high-income countries. Accordingly, the purpose of this analysis is to determine if there are strong data that will support the use of dexamethasone in children with meningitis caused by *S pneumoniae*.

## CAN META-ANALYSES ANSWER THIS QUESTION?

The most recent comprehensive meta-analysis on this topic appeared in 2015 [19]. Brouwer and colleagues, who have been assessing this literature since 2003, concluded that steroids showed a protective effect on severe hearing loss overall (meaning all bacterial etiologies) and a favorable point estimate for severe hearing loss due to non-*Haemophilus* meningitis. Although they favored the use of dexamethasone in all children with meningitis regardless of microorganism, based on a lack of evidence of deleterious effects, they acknowledged that the use of corticosteroids in children other than those with *H influenzae* was still controversial because of the lack of positive evidence of benefit. A frustrating aspect of the review of this meta-analysis is the continued inclusion of studies of very poor quality. By their own evaluation, only 4 of the 25 studies included in the meta-analysis were high quality, 14 were of intermediate quality, and 7 were of poor quality. In addition, they included 9 studies performed in low-income countries in which a positive impact of steroids has never been demonstrated [20].

In search of more recent analyses, we identified the Evidence Review for Corticosteroids for Treatment of Bacterial Meningitis developed by the United Kingdom’s National Institute for Health and Care Excellence and published in 2024 [21]. Their analysis of this topic used Brouwer and colleagues' review and added 2 studies. One large study of 480 patients was conducted in Pakistan [22] and did not indicate the bacterial etiology of the cases reviewed. The other study was conducted in neonates [9]; therefore, neither was contributory to answering our question.

Last, there was a subsequent meta-analysis by Wang et al evaluating only children [23]. This analysis included only 1 additional study not included previously by Brouwer et al but omitted the study by Wald et al [16]. Again, several studies were of poor quality or performed in a low-income country. They concluded that there was a favorable impact of dexamethasone on hearing and neurologic outcome but not mortality. In sum, these meta-analyses, with their shortcomings, are more a commentary on the impact of dexamethasone on meningitis caused by *H influenzae* rather than *S pneumoniae*. To answer our question, we must review the literature on adults with meningitis.

## ADJUVANT STEROIDS IN ADULTS WITH MENINGITIS

Turning to the adult domain where *S pneumoniae* is and has been the dominant cause of acute bacterial meningitis by far, the Infectious Diseases Society of America (IDSA) unequivocally recommended the use of dexamethasone in patients with pneumococcal meningitis in 2004. Their most recent guideline [24] made this recommendation based on a single prospective, placebo-controlled trial reported from Europe in 2002. In that study, the standard antimicrobial therapy for adults with bacterial meningitis was intravenous amoxicillin [25]. Among the patients with pneumococcal meningitis, there were unfavorable outcomes in 26% of the dexamethasone group, as compared with 52% of the placebo group (relative risk, 0.50 [95% confidence interval, .30–.83]; *P* = .006). Fourteen percent of those who received dexamethasone and 34% of those who received placebo died. Although adults who received steroids had fewer neurologic abnormalities including less hearing loss than those who received placebo, these results were not significantly different.

Van de Beek and de Gans [26], who were co-authors of the European study, went on to do a post hoc analysis of the same data set to determine whether the beneficial impact of dexamethasone on mortality reflected its effect on systemic causes or neurologic causes of death. The major impact of steroids appeared to be on the systemic causes of fatality, that is, septic shock and pulmonary complications. This was surprising as steroids have been assessed as adjuvant therapy for the management of patients in septic shock in many studies over the years, with variable results [27]. However, when the same team performed a meta-analysis of individual patient data [28], using data from 5 studies that had been recently published (including their own) in which raw data were available, the authors could not show a reduction of either death or neurologic disability attributable to adjuvant steroids [26, 29–32]. On the surface, these results were unexpected given the previous post hoc analysis, but a closer examination of those studies that were able to contribute raw data is revealing. Of the 5 studies, 2 were reported from the low-income country of Malawi [31, 32], 1 from Vietnam [30], 1 from South America [29], and 1 from Europe [25]. The study from Vietnam did show a benefit of steroids in patients with proven bacterial meningitis [30]. However, in those cases in which there was no identified pathogen (many of which probably had tuberculosis), the recipients of dexamethasone did more poorly. Accordingly, even though the original European study upon which the IDSA made its recommendation was included in this last study and indicated a positive impact of steroids, in 2010 there was not absolute clarity on how or if dexamethasone was beneficial in adult patients with meningitis due to *S pneumoniae*.

**Figure 1.**
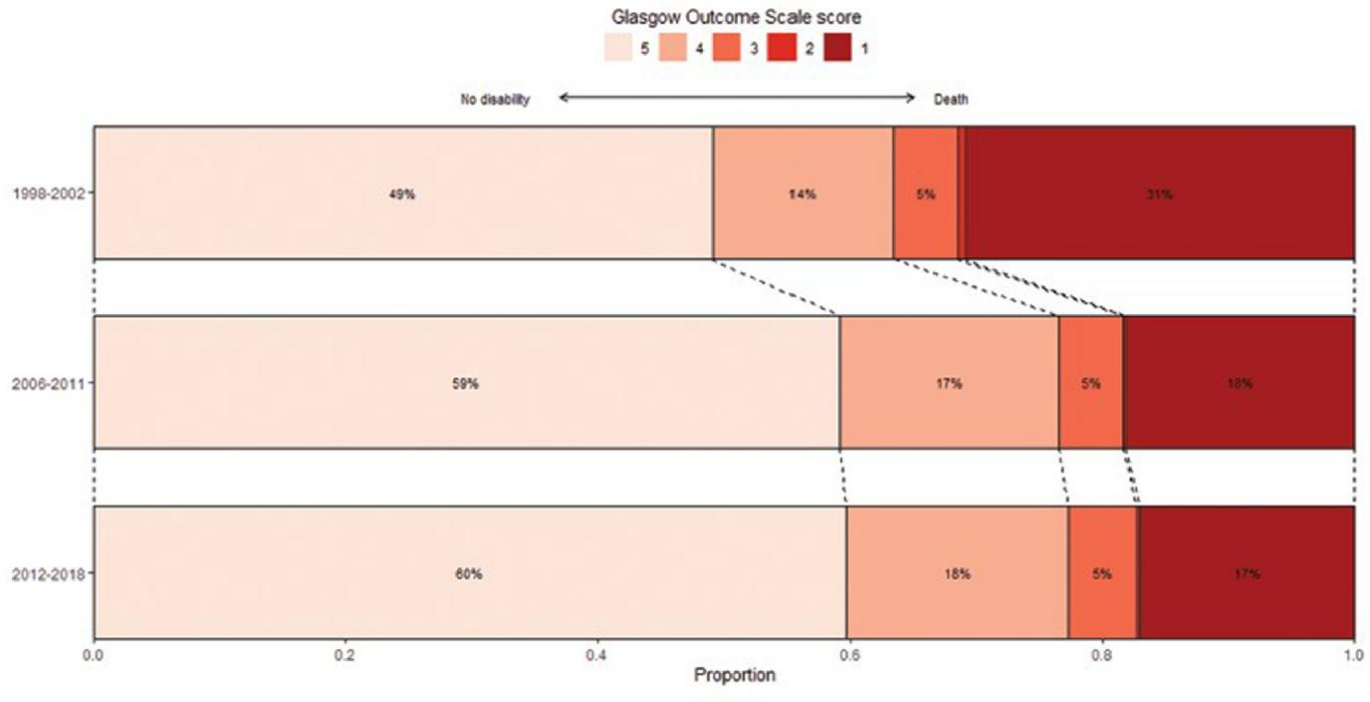
Change in clinical outcome over a 20-year period. Histogram showing the clinical outcome as scored on the Glasgow Outcome Scale (GOS) in 1998–2002, 2006–2011, and 2012–2018. Score 5 indicates mild to no deficits (left), score 1 indicates death (right). Adjunctive dexamethasone was administered between 1992 and 2002 in 1 of 104 patients (1%), 0 of 2 patients (0%), 0 of 17 patients (0%), 1 of 48 patients (2%), and 9 of 165 patients (5%) from GOS 1 to 5, respectively; between 2006 and 2011 in 77 of 108 patients (71%), 1 of 1 patient (100%), 22 of 29 patients (76%), 87 of 104 patients (84%), and 305 of 352 patients (87%) from GOS 1 to 5, respectively; and between 2012 and 2018 in 100 of 141 patients (71%), 2 of 3 patients (67%), 33 of 44 patients (75%), 118 of 148 patients (80%), and 432 of 508 patients (85%) from GOS 1 to 5, respectively. Adapted with permission from Koelman et al [33].

The study that is the most compelling and persuasive is the most recent review of the last 2 decades of pneumococcal meningitis in adults in the Netherlands [33]. The authors reviewed cases of documented pneumococcal meningitis during 3 periods: 1998–2002, 2006–2011, and 2012–2018. They compared outcome according to the 5-point Glasgow Outcome Scale (1, death; 2, vegetative outcome; 3, severe disability [unable to live independently]; 4, moderate disability [unable to work or attend school]; 5, normal or mild disability). Period 1 (1998–2002) was the time during which the European randomized trial was being performed and use of dexamethasone only occurred in 3% of cases. During the next 2 time periods (2006–2011 and 2012–2018), dexamethasone became routine; overall use of dexamethasone was 82% and in 2018 it was 85%. When patients who received dexamethasone were compared to those who did not, recipients of dexamethasone experienced a lower rate of cardiorespiratory failure (323/1156 [28%] vs 237/575 [41%]; *P* < .001), a lower rate of in-hospital seizures (174/1139 [15%] vs 132/569 [23%]; *P* < .001), and a lower rate of focal neurological abnormalities (214/1099 [19%] vs 142/558 [25%]; *P* = .005), leading to a lower rate of poor outcome, defined as ≤4 on the Glasgow Outcome Scale (442/1188 [37%] vs 307/586 [52%]; *P* < .001). It is notable that among the patients with favorable outcome, hearing loss (of unknown severity) was identified in 32%. Dexamethasone was associated with a lower mortality rate both from neurological complications and systemic complications (31% vs 16%). Convincingly, in patients who did not receive dexamethasone according to the protocol, the mortality rate was nearly the same for cohort 1 (32%) and cohort 2 (28%) and for the placebo group (34%) in the original prospective European controlled trial [25]. Figure 1 shows the outcome of patients during these 3 periods.

In further support for dexamethasone is the study from Spain reported by Cabellos et al [34], which describes a 31-year experience using what they describe as low-dose dexamethasone, a single dose of mannitol, and anticonvulsant prophylaxis as standard adjuvant treatment of patients with pneumococcal meningitis. When they compare recipients of dexamethasone to those who did not receive dexamethasone, they demonstrate substantial differences in mortality and overall good clinical outcome. Clearly, their use of anticonvulsant prophylaxis may have contributed to outcome. In addition, the few patients in whom dexamethasone was not used had outcomes comparable to those seen before 1987 when their protocol for triple treatment was introduced.

Finally, in a recent study that reported a quasi-experimental propensity score–based investigation of 30-day all-cause mortality after hospital admission for children with pneumococcal meningitis, the authors reported a reduced risk of death for children who received dexamethasone within 12 hours of receipt of antibiotics [35]. The study encompassed the years 2005–2022 for cases reported to the French National Surveillance System, representing approximately 61% of cases. The adjusted 30-day death rate was 6% versus 12% in the dexamethasone versus no dexamethasone groups, respectively (marginal odds ratio, 0.39 [95% confidence interval, .23–.65]).

## DEXAMETHASONE

Dexamethasone is a potent synthetic steroid that is frequently used for its anti-inflammatory and immunosuppressive capabilities. Despite several decades of clinical use, the precise mechanisms involved in its role as an anti-inflammatory agent are not fully known. In addition to suppressing the migration of neutrophils and lymphocytes, dexamethasone stabilizes vascular endothelial membranes and inhibits the signaling of several cytokines and Toll-like receptors. Although one might ponder the need to question the role of dexamethasone in meningitis caused by *S pneumoniae*, if it is known to be effective in meningitis caused by *H influenzae*, given the differences in the inflammatory reaction stimulated by gram-positive and gram-negative organisms (and *S pneumoniae* and *H influenzae* in particular), the question is meaningful [4].

While adverse reactions associated with prolonged courses of steroids are well-known, adverse reactions associated with short-term, low-dose use include gastrointestinal bleeding, potential thromboses, and potential for the delayed sterilization of the CSF. In reported clinical usage, dexamethasone has appeared safe. Because dexamethasone is associated with adverse reactions, there has been some interest in determining the lowest dose with the greatest effectiveness. Several studies of 2 days rather than 4 days have shown similar effectiveness [17, 34].

As dexamethasone needs to be started very early in the treatment process, there will be the unavoidable initiation of steroids in patients who prove to have viral or aseptic meningitis. In a study of adult patients with viral meningitis, there was no dose-dependent association between dexamethasone and a poor outcome [36]. For enterovirus infections in particular, the odds ratio for an unfavorable outcome did increase when ≥5 doses of dexamethasone were administered. Although a sensitivity analysis indicated that the association was affected by unmeasured or residual confounding by severity, an editorial comment stresses the need to affirm the nonbacterial diagnosis promptly to limit the number of dexamethasone doses [37].

Is there a hazard in using dexamethasone in individuals who prove to have meningitis due to *N meningitidis?* No evidence of a detrimental effect has been documented [38].

## FINAL QUESTION

The final question we must address, to be convinced that the recommendation to use adjuvant steroids in pneumococcal meningitis in children is appropriate, is whether there is reason to believe that the host inflammatory response to infection with *S pneumoniae* or the biologic effect of dexamethasone is different in the age group 2 months to 17 years from that of those ≥18 years of age.

According to Davis and Tuomanen, the molecular mechanisms activated by the host in response to central nervous system infections caused by *S pneumoniae* are similar regardless of age (ie, in infants and the elderly) [39]. In studies of patients with meningitis in which inflammatory mediators have been measured in the CSF, there is no apparent alteration according to age [8, 40]. Most importantly, the anti-inflammatory effect created by dexamethasone is observed across the age continuum when prescribed in many different inflammatory and autoimmune conditions.

## CONCLUSIONS

There are convincing data to support the use of dexamethasone as adjunctive treatment for pneumococcal meningitis in children. When initiating treatment for an infant (beyond the neonatal age group) or child with suspected community-acquired bacterial meningitis, dexamethasone at 0.15 mg/kg/dose every 6 hours for 4 days should be given, starting concurrently or 15 minutes before administration of antibiotics. The number of doses of dexamethasone administered to children proven not to have bacterial meningitis should be limited as soon as possible.
